# Supplementary material for: Metabolomics-Based Analyses of Dynamic Changes in Flavonoid Profiles in the Black Mulberry Winemaking Process
Source: Foods. 2023 May 31;12(11):2221. doi: 10.3390/foods12112221 (PMC10252360; doi:10.3390/foods12112221)
Supplement: Supplementary file 1 [file foods-12-02221-s001.zip › Supplementary data2.pdf]

## Supplementary Materials

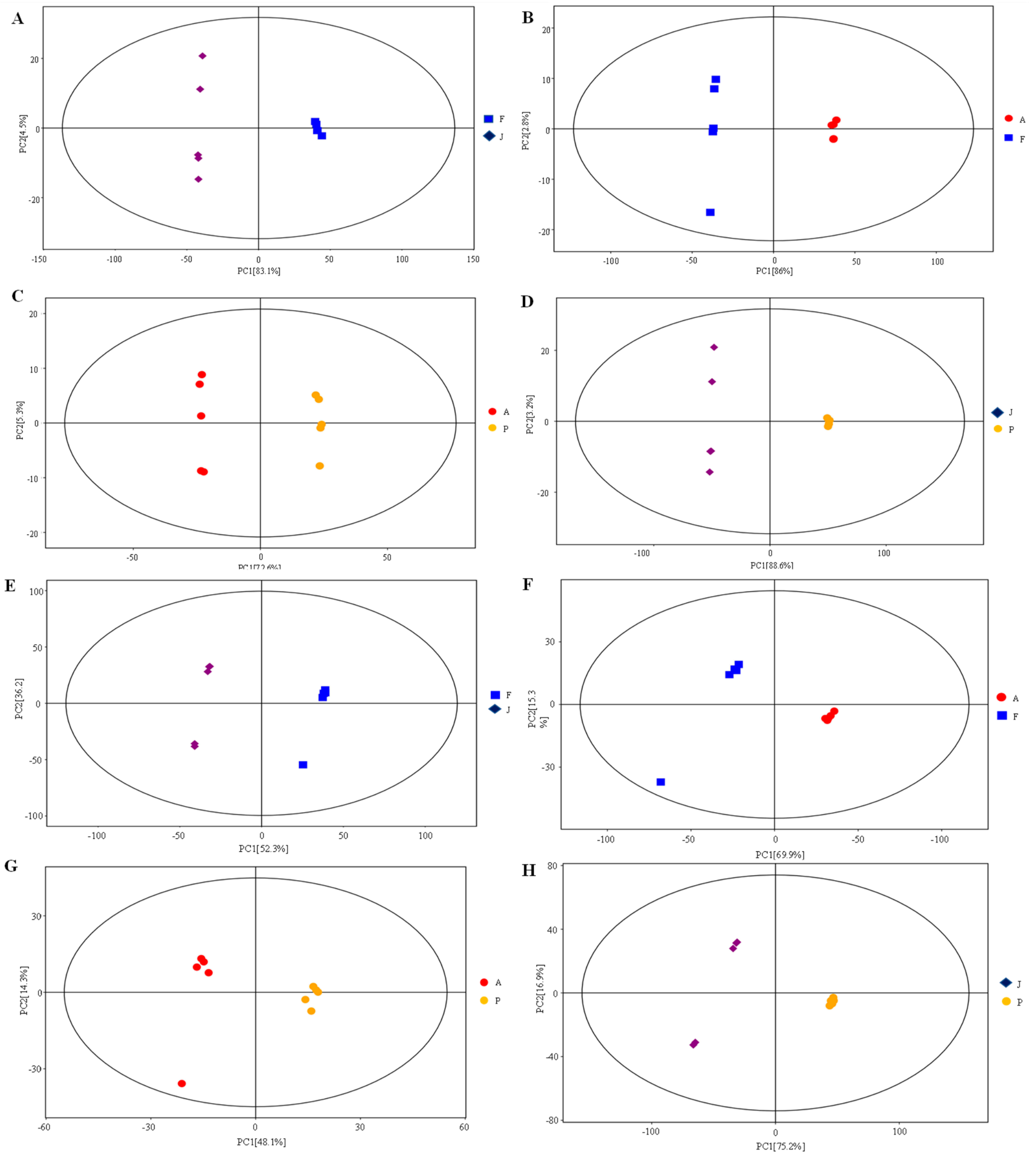

**Figure S1**. Plot of PCA scores between different stages in ESI$^+$(**A–D**) and ESI$^-$(**E–H**). (Purple: J stage; Blue: F stage; Red: A stage; Orang: P stage)

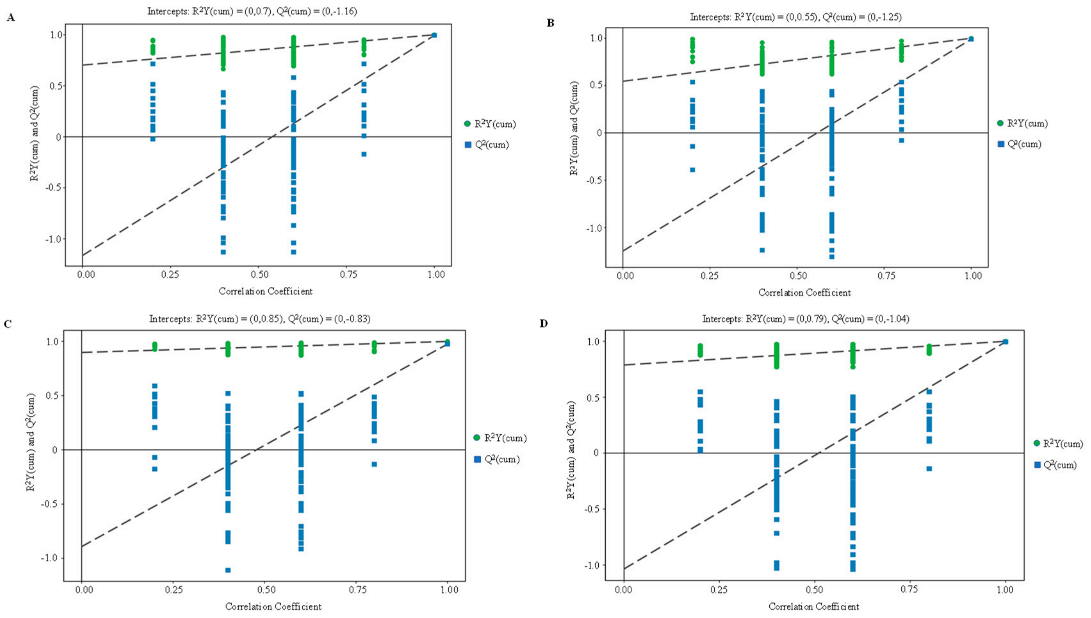


**Figure S2**. Cross-Validation and Alternate Testing of OPLS-DA Models (ESI$^+$). ((**A**): J vs F; (**B**): F vs A; (**C**): A vs P; (**D**): J vs P).

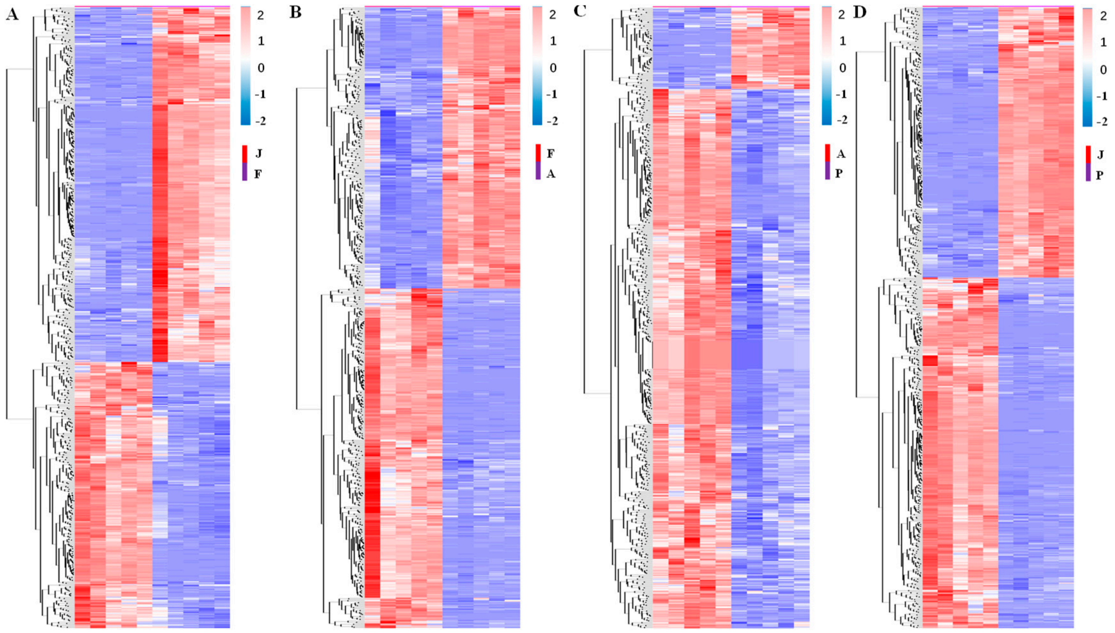


**Figure S3.** Hierarchical cluster analysis heat map of different brewing stages of mulberry wine. The sample names are displayed in the abscissa, and the differential metabolites are displayed in the ordinate. The color transformation from blue to red represents the relative abundance of metabolite expression from low to high. ((**A**): J vs F; (**B**): F vs A; (**C**): A vs F; (**D**): J vs P).

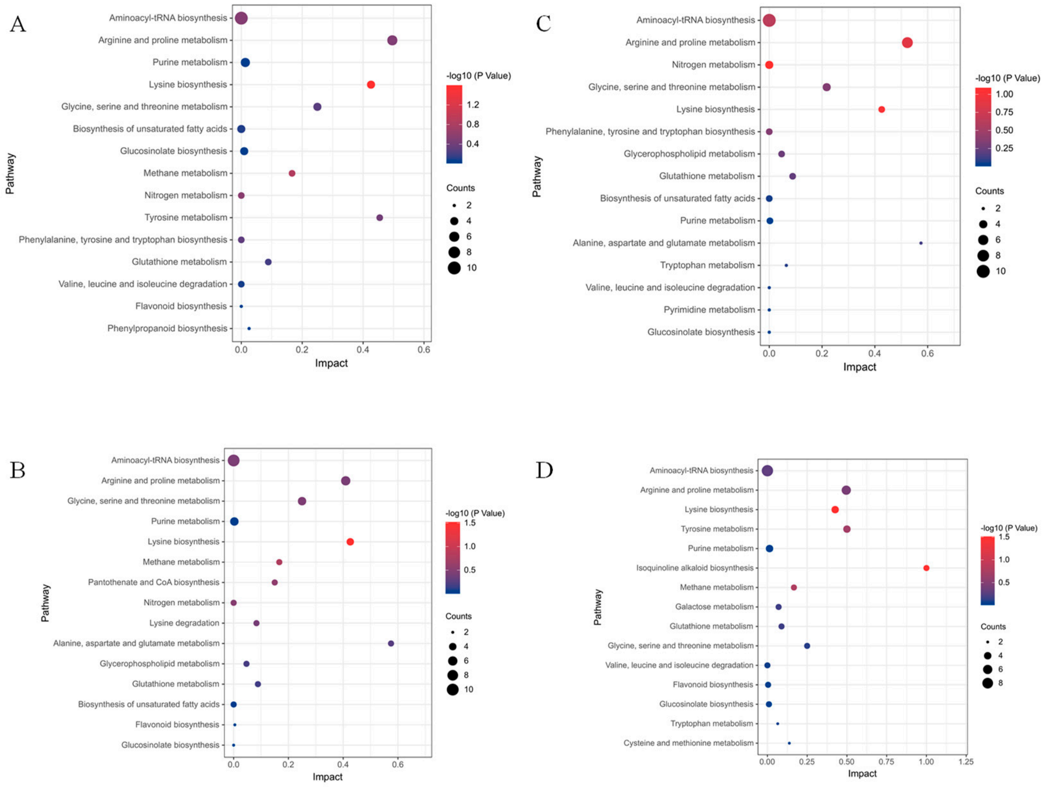


**Figure S4.** Enrichment analysis of differential metabolite pathways ((**A**): J vs F; (**B**): F vs A; (**C**): A vs P; (**D**): J vs P). The color represents the p-value and the bubble represents the number of enrichments of differential metabolites.

**Supplementary Table S2.** Mantel test result of the comparisons between major metabolites and physicochemical factors in mulberry wine brewing process.

| Differential Metabolites | Physicochemical Factors | Mantel's r |
|---|---|---|
| Amino acids | pH | 0.72 |
| | TS | 0.76 |
| | TSS | 0.21 |
| | TA | 0.29 |
| | Alcohol | 0.86 |
| Aromatic compounds | pH | 0.82 |
| | TS | 0.87 |
| | TSS | 0.18 |
| | TA | 0.50 |
| | Alcohol | 0.92 |
| Fatty acids | pH | 0.43 |
| | TS | 0.51 |
| | TSS | 0.52 |
| | TA | 0.22 |
| | Alcohol | 0.63 |
| Organic acids | pH | 0.59 |
| | TS | 0.69 |
| | TSS | 0.41 |
| | TA | 0.21 |
| | Alcohol | 0.77 |
| Polyphenols | pH | 0.49 |
| | TS | 0.58 |
| | TSS | 0.45 |
| | TA | 0.17 |
| | Alcohol | 0.68 |
| Carbohydrates | pH | 0.11 |
| | TS | 0.20 |
| | TSS | 0.73 |
| | TA | 0.12 |
| | Alcohol | 0.33 |
